# Systematic review of humeral shaft fracture (OTA/AO 12) complicated with iatrogenic radial nerve injury

**DOI:** 10.1186/s40001-024-01981-7

**Published:** 2024-07-25

**Authors:** Zeyu Zhang, Zhongpei Lin, Qinglin Qiu, Xincai Xiao, Shouwen Su, Xiaoyue Wen, Bo He

**Affiliations:** Joint and Orthopaedic Trauma, Department of Orthopedics, The Third Affiliated Hospital of Sun Yat-Sen University, No. 600 Tianhe Road, Guangzhou, Guangdong China

**Keywords:** iRNI, Humerus, Fracture, Systematic review

## Abstract

**Objectives:**

To compare the iatrogenic radial nerve injury (iRNI) rate of different implant (plate *vs.* intramedullary nail) and surgical approaches during humeral shaft fracture surgery.

**Methods:**

The online PubMed database was used to search for articles describing iRNI after humeral fracture with a publication date from Jan 2000 to October 2023. The following types of articles were selected: (1) case series associating with adult humeral shaft fracture, preoperative radial nerve continuity, non-pathological fracture and non-periprosthetic fracture; (2) involving humeral shaft (OTA/AO 12) fractures. Articles where we were unable to judge surgical approach or fracture pattern (OTA/AO 12) were excluded. The data were analyzed by SPSS 27.0 and Chi-square test was performed to identify incidence of iRNI associated with different implant and surgical approaches.

**Results:**

Fifty-four articles with 5063 cases were included, with 3510 cases of the plate, 830 cases of intramedullary nail and 723 cases of uncertain internal fixation. The incidences of iRNI with plate and intramedullary nail were 5.95% (209/3510) and 2.77% (23/830) (*p* < 0.05). And iRNI incidences of different surgical approaches were 3.7% (3/82) for deltopectoral approach, 5.74% (76/1323) for anterolateral approach, 13.54% (26/192) for lateral approach and 6.68% (50/749) for posterior approach. The iRNI rates were 0.00% (0/33) for anteromedial MIPO, 2.67% (10/374) for anterolateral MIPO and 5.40% (2/37) for posterior MIPO (*p* > 0.05). The iRNI rates were 2.87% (21/732) for anterograde intramedullary nail and 2.04% (2/98) for retrograde intramedullary nail (*p* > 0.05). In humeral bone nonunion surgery, the rate of iRNI was 15.00% (9/60) for anterolateral approach, 16.7% (2/12) for lateral approach and 18.2% (6/33) for posterior approach (*p*  > 0.05).

**Conclusion:**

Intramedullary nailing is the preferred method of internal fixation for humeral shaft fractures that has the lowest rate of iRNI. Compared with anterolateral and posterior approaches, the lateral surgical approach had a higher incidence of iRNI. The rate of iRNI in MIPO was lower than that in open reduction and internal fixation.

**Level of evidence:**

Level IV.

## Introduction

Humeral shaft fractures account for about 1–5% of all fractures [1–4] and 20% of humeral fractures [5]. A considerable part of humeral shaft fractures needs surgical treatment, and iatrogenic nerve injury may occur during operation. A single-center retrospective study by Entezari et al. found that the incidence of humeral shaft fracture combined with iatrogenic nerve injury was 4.6% (7/154), all of which involved the radial nerve [6]. The incidence of humeral shaft fractures complicated with radial nerve injury varies in the literature. The retrospective study by Claessen et al. of 6 hospitals found that the incidence of humeral shaft fracture complicated with iRNI was about 7% (18/259) [7].

It is reported that the incidence of iRNI is different with different implants and different surgical approaches. Amer et al. reviewed 3 studies and reported that the incidence of nerve injury was 10.8% (12/111) for open reduction and internal fixation with plate and 0% (0/104) for intramedullary nail [8]. A retrospective study by Claessen et al. on 6 hospitals found that the corresponding probabilities of nerve injury were 4% (7/165) for anterolateral approach, 22% (2/9) for lateral approach and 11% (9/85) for posterior approach [7].

This paper reviews the literature on humeral shaft fractures complicated by iatrogenic radial nerve injury from Jan 2000 to October 2023 in the PubMed database to investigate the relationship between the implant (plate *vs*. intramedullary nail), surgical approach, and iRNI, to assist clinicians in selecting implants and surgical approach.

## Methods

### Literature retrieval

**Fig. 1:**
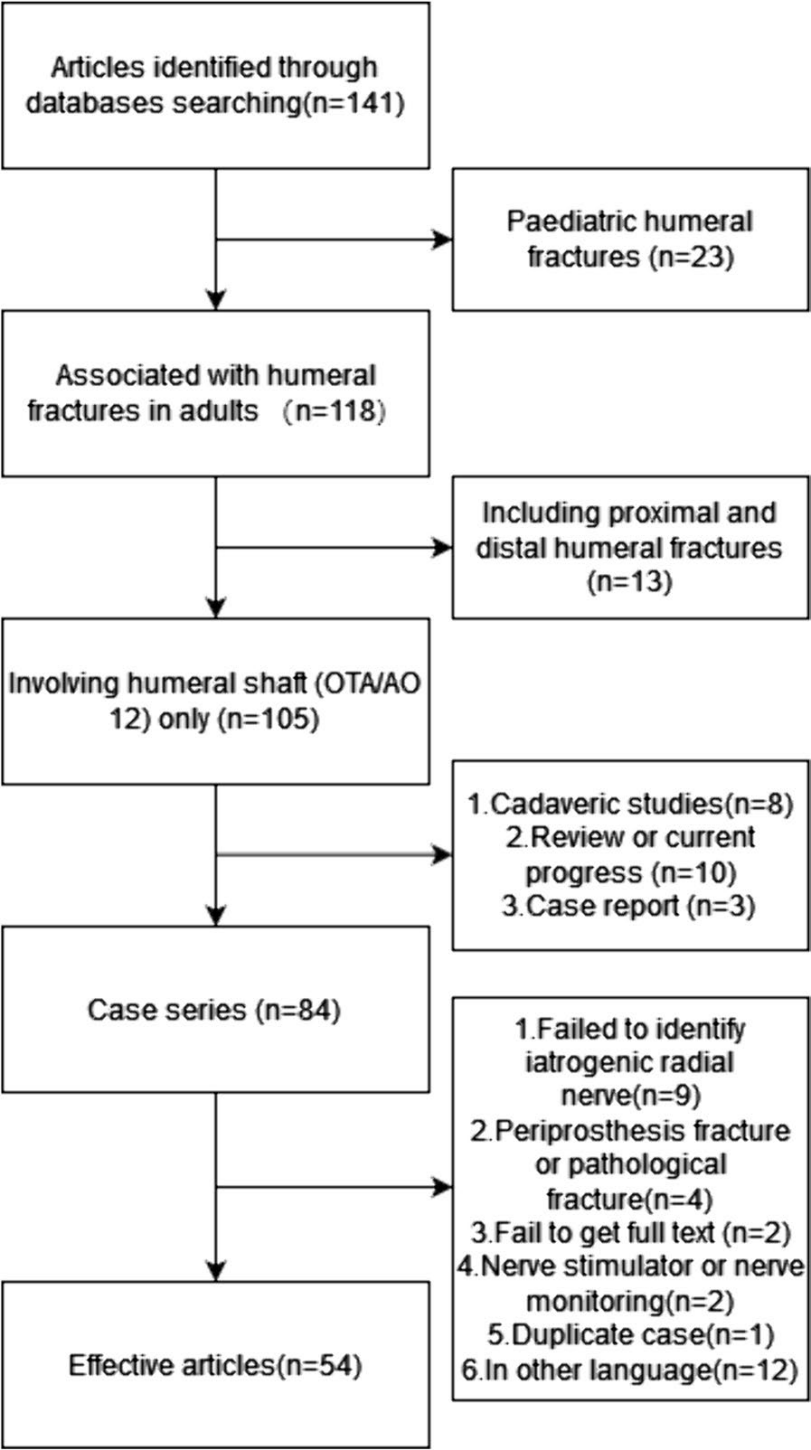
Study identification and selection process

The literatures on humeral fracture (OTA/AO 11/12/13 A/B/C [9]) combined with radial nerve injury was retrieved in PubMed with "(humeral fracture) AND (iatrogenic radial nerve injury)" as the search term, with a publication date from Jan 2000 to October 2023. Details of the study identification and selection process are summarized in Fig. 1.

### Inclusion, exclusion and rejection criteria

The inclusion criteria were: (1) full text documents; (2) adult patients; (3) preoperative radial nerve continuity; (4) non-pathological fracture; (5) non-periprosthetic fracture; (6) humeral shaft fracture (OTA/AO 12).

The exclusion criteria were: (1) no available full text; (2) inability to judge surgical approach.

The rejection criteria were: (1) misinclusion; (2) unable to judge whether the humeral shaft was involved (OTA/AO 12); (3) fractures combined with proximal humerus (OTA/AO 11) or distal humerus (OTA/AO 13).

### Quality evaluation

In accordance with the above criteria, there were two researchers searching the literature independently. On the basis of titles and abstracts, all articles which were not associated with our study were excluded. When abstracts provided insufficient information, the full articles were reviewed. The results from different researchers were carefully compared. In terms of different opinions, the final decisions were made by a third researcher after discussion regarding inclusion of the papers.

### Intervention factors and statistical analysis

The data on implant types and surgical approaches were extracted from the literature. Data used for comparison and analysis included: 1. implant types (plate and intramedullary nail); 2. deltopectoral approach, anterolateral approach, lateral approach and posterior approach of plate [10, 11]; 3. anteromedial [12, 13], anterolateral approach and posterior approach of minimally invasive plate osteosynthesis (MIPO); 4. anterograde and retrograde intramedullary nail.

The data were analyzed by SPSS 27.0 (SPSS, Chicago, IL, USA), and the qualitative data were tested by Chi-square test. The significance level was set to 0.05. When comparing multiple groups with each other, Bonferroni correction was used. Fisher precise test was used when necessary.

## Results

A total of 141 studies were retrieved, and finally 54 effective studies were included after manual screening.

### Comparison of iRNI with different implants (plate and intramedullary nail)

A total of 54 articles were included, including 48 articles about plates and 14 articles about intramedullary nail. The total number of cases was 5063, including 3510 cases of the plate, 830 cases of intramedullary nail and 723 cases of uncertain internal fixation.

The rate of iRNI combined with humeral shaft fracture was 7.54% (278/5063). The rate of iRNI with plate and intramedullary nail was 5.95% (209/3510) and 2.77% (23/830), respectively ($\chi^{2}$ = 13.444, *p* < 0.001) (Table 1).

**Table 1:** Comparison of iatrogenic radial nerve injury caused by bone plate VS intramedullary nail

	Injury	Non-injury	Chi-square value	*p* value
Bone plate	209(6.0%)	3301(94.0%)	13.444	< 0.001
Intramedullary nail	23(2.8%)	807(97.2%)		

### Comparison of iRNI by different surgical approaches (excluding MIPO)

Twenty-four articles about open reduction and internal fixation with plate with defined approaches were included, including 2 on deltopectoral approach, 14 on anterolateral approach, 5 on lateral approach and 13 on posterior approach. The total number of cases was 2346, including 82 cases of deltopectoral approach, 1323 cases of anterolateral approach, 192 cases of lateral approach and 749 cases of posterior approach.

In cases of open reduction and internal fixation with a plate with a defined surgical approach (excluding MIPO), the incidence of radial nerve injury was 6.61% (155/2346). The corresponding incidences were 3.7% (3/82) for deltopectoral approach, 5.74% (76/1323) for anterolateral approach, 13.54% (26/192) for lateral approach and 6.68% (50/749) for posterior approach (Table 2). There were statistical differences between anterolateral approach and lateral approach (5.74% vs 13.54%, $\chi^{2}$ = 16.233, *p* < 0.001), and between lateral approach and posterior approach (13.54 vs 6.68%, $\chi^{2}$ = 9.704, *p* = 0.002) (α = 0.008 after Bonferroni correction).

**Table 2:** Comparison of iatrogenic radial nerve injuries of deltopectoral, anterolateral, lateral and posterior approaches (excluding MIPO)

	Injury	Non-injury	Chi-square value	*p* value
Deltopectoral	3(3.7%)	79(96.3%)	17.719	< 0.001
Anterolateral	76(5.7%)	1247(94.3%)		
Lateral	26(13.5%)	166(86.5%)		
Posterior	50(6.7%)	699(93.3%)		

There were 15 studies without a definite approach of plate operation, and the incidence of radial nerve injury was 5.90% (42/712) in this subset, which was not included in the statistics.

### Comparison of iRNI caused by plate internal fixation, intramedullary nail, and MIPO

Fifty-three articles were included, where plates were involved in 38 articles, intramedullary nails were involved in 14 articles, and MIPO was involved in 17 articles. The total number of cases was 4340, including 3058 cases of plate fixation, 830 cases of intramedullary nail and 452 cases of MIPO. The injury rates of radial nerve were 6.44% (197/3058), 2.77% (23/830) and 2.65% (12/452), respectively. There were statistical differences between plate fixation and intramedullary nail (6.44% *vs* 2.77%, $\chi^{2}$ = 16.480, *p* < 0.001), plate fixation and MIPO (6.44% *vs* 2.65%, $\chi^{2}$ = 10.086, *p* < 0.001), but no difference between an intramedullary nail and MIPO ($\chi^{2}$ = 0.015, *p* = 0.903) (α = 0.017 after Bonferroni correction) (Table 3).

**Table 3:** Comparison of iatrogenic radial nerve injury caused by open reduction and plate fixation, intramedullary nail and MIPO

	Injury	Non-injury	Chi-square value	*p* value
Plate fixation	197(6.4%)	2861(93.6%)	24.607	< 0.001
Intramedullary nail	23(2.8%)	807(97.2%)		
MIPO	12(2.7%)	440(97.3%)		

### Comparison of iRNI caused by MIPO through different surgical approaches

Sixteen articles were included, including 2 articles of anteromedial MIPO, 13 articles of anterolateral MIPO, and 1 article of posterior MIPO. The total number of cases was 444, including 33 cases of anteromedial MIPO, 374 cases of anterolateral MIPO and 37 cases of posterior MIPO. The radial nerve injury rates were 0.00% (0/33), 2.67% (10/374) and 5.40% (2/37). There was no significant difference among them ($\chi^{2}$ = 1.654, *p* = 0.432, Fisher–Freeman–Halton precise test) (Table 4).

**Table 4:** Comparison of anteromedial, anterolateral and posterior MIPO iatrogenic radial nerve injuries

	Injury	Non-injury	Chi-square value	*p value*
Anteromedial MIPO	0 (0.0%)	33 (100%)	1.654	0.432
Anterolateral MIPO	10 (2.7%)	364 (97.8%)		
Posterior MIPO	2 (5.4%)	35 (94.6%)		

A retrospective study by Huang et al. proposed an anterolateral double-incision approach. The proximal incision was made at the median anterior humerus, the biceps brachii was pulled medially, and the lateral third of brachialis was split to expose the fracture position. The distal incision was made between brachioradialis and triceps [14]. In this paper, we included it in the anterolateral MIPO approach.

### Comparison of iRNI caused by intramedullary nail between different approaches

Fourteen articles were included here, including 12 articles on anterograde intramedullary nail and 3 articles on retrograde intramedullary nail. The total number of cases was 830, including 732 cases of anterograde intramedullary nail and 98 cases of retrograde intramedullary nail. The proportion of radial nerve injury was 2.87% (21/732) and 2.04% (2/98), respectively, and there was no significant difference between them (*p* = 1.000, Fisher precise test) (Table 5).

**Table 5:** Comparison of iatrogenic radial nerve injury caused by anterograde and retrograde intramedullary nail

	Injury	Non-injury	*p* value(Fisher)
Anterograde	21(2.9%)	711(97.1%)	1.000
Retrograde	2(2.0%)	96(98.0%)	

### Comparison of iRNI in humeral nonunion patients among different approaches

There were 3 articles about nonunion, including 1 article on intramedullary nail and 3 articles on plates. Among them, 1 article was about anterolateral/deltopectoral approach. There were 129 cases, including 24 cases of intramedullary nail and 105 cases of plate, where 60 cases were anterolateral approach, 12 cases were lateral approach, and 33 cases were posterior approach. All intramedullary nails were retrograde and there were no iatrogenic nerve injuries. The rate of iRNI was 15.0% (9/60) in cases of anterolateral approach, 16.7% (2/12) in cases of lateral approach and 18.2% (6/33) in cases of posterior approach. There was no significant difference among them ($\chi^{2}$ = 0.336, *p* = 0.928, Fisher–Freeman–Halton precise test) (Table 6).

**Table 6:** Comparison iatrogenic radial nerve injuries in humeral nonunion patients among different approaches

	Injury	Non-injury	Chi-square value	*p* value
Anterolateral	9 (15.0%)	51 (85.0%)	0.336	0.928
Lateral	2 (16.7%)	10 (83.3%)		
Posterior	6 (18.2%)	27 (81.8%)		

A set of data in Oliver et al.'s study was not included in the statistics because we could not distinguish if it used the anterolateral approach or deltopectoral approach [15].

## Discussion

The overall age distribution of patients with humeral shaft fracture was a bimodal pattern, with a small peak at 21–30 years old and a large peak at 71–80 years old [16]. The main groups were younger patients suffering from high-energy trauma and older patients suffering from low-energy trauma [4]. Entezari et al. found that distal-third humeral shaft fractures (*p* < 0.05, OR 6.3), high-energy trauma (*p* < 0. 05, OR 1.7), open fracture (*p* < 0. 05, OR 2.1) and concomitant vascular injury (*p* < 0.05, OR 26.9) were independent predictive factors of primary nerve injury [6].

Peripheral nerve injury is one of the common complications of humeral shaft fracture in the early stage. A prospective study by James et al. on more than 5,700 patients with multiple injuries suggested that radial nerve injury was the most common peripheral nerve injury, and 9.5% of humeral fractures were complicated by radial nerve injury [17]. Niver et al. found that the rate of humeral shaft fractures associated with radial nerve injury was about 2–17% [10, 18–20]. Shao et al. found in their retrospective study that the rate of humeral shaft fracture complicated with radial nerve injury was 11.8% (532/4517), and middle and distal humeral shaft fractures were more likely to damage the radial nerve [21]. However, Ljungquist et al. mentioned that the rate of radial nerve injury caused by humeral shaft fracture was 22% [22], and Streufert et al. summarized the case data of two centers and found that the incidence of radial nerve injury was 18.4% (48/261) [23]. Entezari et al. reported that the incidence of humeral shaft fracture complicated by nerve injury was about 25.5% (96/376), among which radial nerve injury accounted for about 94%, and the iatrogenic nerve injury incidence rate of humeral shaft fracture was 4.6% (7/154), all of which involved the radial nerve [6]. In our study, the total rate of iRNI in humeral shaft fracture was 7.54% (278/5063), and there was no significant statistical difference with Entezari’s reports (7.54% vs 4.6% *p* > 0.05).

### Comparison of iRNI with different implants (plate and intramedullary nail)

The iatrogenic injury rates of the radial nerve caused by different treatment methods are different. For example, it has been reported that the iatrogenic injury rate of radial nerve of plate was 6.5%–12.0%, and that of intramedullary nails was 2.7–5.0% [4, 24, 25]. This is similar to our statistical results, plate of 5.95% (209/3510), intramedullary nail of 2.77% (23/830). Amer et al. included three pieces of literatures in their meta-analysis study, and the results showed that for humeral shaft fracture (OTA/AO 12), plate (10.8%, 12/111) was more likely to cause iRNI than intramedullary nail (0%, 0/104) (*p* < 0.05) [8]. However, Streufert et al. reported that the two-center study from 2008 to 2016 suggested that the iRNI rate of the plate was 15.6% (10/64) in the middle humeral shaft and 15% (16/107) in the distal humeral shaft [23], which may be related to the diagnosis and treatment level of medical centers.

Ouyang et al. conducted a meta-analysis on 10 randomized controlled trials (RCT) and 439 cases and found that compared with intramedullary nail, plate avoided the risk of postoperative acromion impingement and limited shoulder joint movement, but there was no significant difference in iRNI (*p* > 0.05) [26]. Kurup et al. reviewed five low-quality (unstratified) randomized trials, involving 260 participants, and reached the same conclusion as Ouyang et al. [27].

We included 3 articles about nonunion, including 1 article about intramedullary nails and 3 articles about plates. Martínez et al. studied the treatment of middle and upper 2/3 of humeral shaft nonunion and found that the rate of iRNI in non-reamed intramedullary nail and bone graft group was lower than that in open reduction and plate internal fixation group (0%, 0/24 *vs*. 11.54%, 3/26) [28]. However, Singh et al. studied humeral shaft nonunion and found that there was no difference between intramedullary nail and plate internal fixation by posterior approach in nonunion healing time, joint function and iRNI rate (0%, 0/20 *vs*. 10%, 2/20) [29]. Koh et al. studied 379 cases of humeral shaft nonunion in 18 trauma centers, and found that there was no difference among anterolateral, lateral and posterior approaches (triceps-reflecting and triceps-splitting) approach, and fracture location (middle humerus) was the only related factor of radial nerve injury (*p* < 0. 05) [30]. Olarte et al. found that radial nerve transposition was an effective auxiliary means for internal fixation of humeral shaft fracture, which was suitable for high-risk nonunion patients [31]. A retrospective study of 19 patients by Chamseddine et al. found that medial transposition of the radial nerve was a safe and reliable method for patients who needed to take out internal fixation and nonunion [32].

Zalavras et al. provided a protocol for the treatment of nonunion of humeral shaft fracture and reported that 41 cases of nonunion were healed within 17 years without iRNI; the protocol was also applicable to patients with long-term nonunion caused by complications [33].

### Comparison of iRNI by different surgical approaches (excluding MIPO)

Surgical approaches for humeral fractures included deltopectoral approach [34], anteromedial approach [12, 13], anterolateral approach, lateral approach and posterior approach. The posterior approach included triceps-reflecting approach (Bryan–Morrey) [35], modified triceps-reflecting approach (Gerwin/Hotchkiss/Weiland) [36], triceps-reflecting anconeus pedicle approach (TRAP) [37], triceps-on approach/paratricipital approach (Alonso-Llames), triceps-splitting approach, the chevron olecranon V osteotomy, and tongue-shaped flap approach, etc.

Surgical approaches for humeral shaft fractures mainly included anterolateral, lateral and posterior approaches, and occasionally deltopectoral approach [34]. Anteromedial approach was only seen in two pieces of literatures for MIPO treatment of humeral shaft fractures [12, 13].

A retrospective study of 6 hospitals by Claessen et al. found that the rate of iRNI caused by humeral shaft fracture was 7% (18/259); the surgical approach was related to iRNI (p < 0.05), which was the only related factor (OR = 6.4 > 1); the incidence of iRNI was 4% (7/165) in the anterolateral approach, 22% (2/9) in the lateral approach, and 11% (9/85) in the posterior approach [7]. In our results, The rate of iRNI in humeral shaft fracture was 7.54% (278/5063), which was similar to the results of Claessen (anterolateral: 5.74%,76/1323; lateral: 13.54%, 26/192; *p* > 0.05); while the rate of radial nerve injury in Posterior approach was 6.68% (50/749), which was lower than that of Claessen 11% (9/85) (*p* < 0.05). However, in our study, there were statistical differences between anterolateral approach and lateral approach (*p* < 0.05), and between the lateral approach and posterior approach in the rate of iRNI (*p* < 0.05).

Multiple regression analysis by Shoji et al. showed that iRNI had nothing to do with surgical approach and timing, but it was related to distal humeral fracture (AO/OTA 12A/B/C) and secondary or multiple operations due to a previous fracture [38].

Streufert et al. analyzed the case data of two centers and found that there was no difference in iRNI rate among the three approaches: which were anterior/anterolateral approach (6/84), triceps lifting approach (14/78) and triceps-splitting approach (6/51) (*p* > 0.05) [23]. A meta-analysis conducted by Shon et al. on 1303 cases in 9 studies showed that the rate of iRNI through posterior approach (13.88%, 69/497) was significantly higher than that through anterolateral approach (5.16%, 35/687) (OR 2.72; 95% CI 1.70–4.35; *p* < 0.05) [39]. There was no significant difference between posterior approach and anterolateral approach (5.74%, 76/1323 *vs* 6.68%, 50/749 *p* > 0.05).

### Comparison between open reduction and internal fixation (plate and intramedullary nail) and MIPO in iRNI

A review by Tetsworth et al. suggested that MIPO had a lower rate of iRNI [40]. A meta-analysis of two randomized controlled trials and three non-randomized controlled trials by Yu et al. found that the rate of iRNI in patients with MIPO was lower than that of traditional open reduction and internal fixation (*p* < 0. 05) [41]. A meta-analysis by Hu et al. included 391 patients and 8 studies, involving 4 randomized controlled trials (RCTs), 2 prospective cohort trials and 2 retrospective cohort trials [42]. It was also found that the iRNI rate in the MIPO group was lower than that in open reduction and internal fixation, and the adjacent joint function score of MIPO was higher than that of intramedullary nail (IMN) (*p* < 0.05). Zhao et al. obtained the same results in a network meta-analysis of 16 randomized controlled trials (OR, 11.09; 95% CI 1.80–124.20) [43]. However, they also found that the incidence of acromion impingement in the intramedullary nail group was higher than that in the open reduction and plate internal fixation group (OR, 0.13; 95% CI 0.03–0.37) and MIPO group (OR, 0.08; 95% CI 0.00–0.69). There were no differences between intramedullary nails, open reduction and plate internal fixation, and MIPO in delayed union, nonunion and infection of humeral fractures [43].

Our results showed that the rate of iRNI in MIPO was lower than that in open reduction and internal fixation (2.65%, 12/452 *VS* 6.44%, 197/3058) (*p* < 0.05), which was consistent with the mentioned literatures.

### Comparison of iRNI caused by MIPO through different surgical approaches

At present, the main surgical approaches for the treatment of humeral shaft fractures by MIPO are anterolateral or posterior. However, Yang et al. found that medial MIPO was a safe surgical method for extra-articular fractures of the middle and distal humerus (0%, 0/12) [12]. A single-center retrospective study by Liu et al. found that the radial nerve injury rate of anteromedial MIPO was low (0%, 0/21). The median nerve, ulnar nerve and brachial artery were protected by brachial muscle through the subbrachial tunnel without injury [13].

The results of this study showed that there was no significant difference among anterolateral MIPO, anteromedial MIPO and posterior MIPO in the rate of iRNI (*p* > 0.05).

### Comparison of iRNI caused by an intramedullary nail through different approaches

There are two methods of intramedullary nailing for humeral shaft fracture: anterograde nail and retrograde nail. In this review, we found that there was no statistical difference in the incidence of iRNI between anterograde and retrograde nailing (*p* > 0.05). Li et al. also found in their retrospective study that there was no significant difference in iRNI rate between anterograde and retrograde intramedullary nailing [44].

### Selection and optimization of implants

Wang et al. found that pre-contouring plates on 3D-printed fracture models can better help young doctors complete operations [45]. For transverse fractures, 6-hole or 7-hole plates are generally used. However, clinically, most fractures are short oblique or long oblique fractures, or even comminuted fractures. Therefore, 8–10 hole plates are often used in clinics. Taking a 10-hole plate as an example, the anatomical study of Chirattikalwong et al. on 56 humerus found that when a 4.5-mm 10-hole compression plate was used for fixation of the middle humeral shaft fracture through an anterolateral approach, the radial nerve would be damaged by the second to sixth holes, and the fourth hole and the fifth hole had the highest rate of injuries [46].

The anatomical study of Chuaychosakoon et al. on 18 upper limbs also held that when the middle humeral shaft fracture was fixed with a 4.5-mm 10 holes compression plate through an anterolateral approach, the radial nerve and/or deep brachial artery would be damaged by the second to fifth holes, while the fourth and fifth holes were most likely to damage them, with the injury ratios of 100% and 66.7%, respectively. The relative ratio of the distance between the fourth hole and the lateral epicondyle of the humerus to the length of the humerus was 0.56. The author suggested that a single-layer cortical screw should be used at the fourth hole [47].

An anatomical study by Noger et al. found that when distal interlocking fixation was performed with non-reamed intramedullary nails through an anterograde approach, the medial and lateral locking nails in the middle easily damaged the radial nerve, median nerve, ulnar nerve and brachial artery. The author believed that using the two anteroposterior screws was safe. If you want to improve the locking stability, it is recommended to complete the internal and external locking of the middle screw holes under direct vision [48]. A retrospective study by Helm et al. found that anterograde intramedullary nail combined with cerclage can reduce the incidence of nonunion (2.6%, 2/78) without increasing the incidence of iRNI (4.59%, 5/109) [49].

## Conclusion

Intramedullary nailing is the preferred method of internal fixation for humeral shaft fractures that has the lowest rate of iRNI. Compared with anterolateral and posterior approaches, the lateral surgical approach had a higher incidence of radial nerve injury, so it is highly recommended that a senior surgeon perform this approach. The rate of iRNI in MIPO was lower than that in open reduction and internal fixation.

## Data Availability

All datasets used can be accessed on Pubmed

## References

[1] Cole PA, Wijdicks CA. The operative treatment of diaphyseal humeral shaft fractures. Hand Clin. 2007;23(4):437–48. 18054671 10.1016/j.hcl.2007.11.004

[2] Mann RJ, Neal EG. Fractures of the shaft of the humerus in adults. South Med J. 1965;58:264–8. 14261543 10.1097/00007611-196503000-00002

[3] Balfour GW, Mooney V, Ashby ME. Diaphyseal fractures of the humerus treated with a ready-made fracture brace. J Bone Joint Surg. 1982;64(1):11–3. 7054192 10.2106/00004623-198264010-00002

[4] Pidhorz L. Acute and chronic humeral shaft fractures in adults. Orthop Traumatol Surg Res. 2015;101(1 Suppl):S41-49. 25604002 10.1016/j.otsr.2014.07.034

[5] Rose SH, Melton LJ, Morrey BF, Ilstrup DM, Riggs BL. Epidemiologic features of humeral fractures. Clin Orthop Relat Res. 1982;168:24–30. 10.1097/00003086-198208000-00003 7105548

[6] Entezari V, Olson JJ, Vallier HA. Predictors of traumatic nerve injury and nerve recovery following humeral shaft fracture. J Shoulder Elbow Surg. 2021;30(12):2711–9. 33964428 10.1016/j.jse.2021.04.025

[7] Fm C, Rm P, Do V, Dl H, R D. Factors associated with radial nerve palsy after operative treatment of diaphyseal humeral shaft fractures. J Shoulder Elbow Surg. 2015;24(11):e307–11. 26341025 10.1016/j.jse.2015.07.012

[8] Amer K, Kurland A, Smith B, Abdo Z, Amer R, Vosbikian M, et al. Intramedullary nailing versus plate fixation for humeral shaft fractures: a systematic review and meta-analysis. Arch Bone Jt Surg. 2022;10(8):661–7. 36258745 10.22038/ABJS.2021.59413.2947 PMC9569144

[9] Meinberg EG, Agel J, Roberts CS, Karam MD, Kellam JF. Fracture and dislocation classification compendium-2018. J Orthop Trauma. 2018;32(Suppl 1):S1–170. 29256945 10.1097/BOT.0000000000001063

[10] Niver GE, Ilyas AM. Management of radial nerve palsy following fractures of the humerus. Orthop Clin North Am. 2013;44(3):419–24. 23827843 10.1016/j.ocl.2013.03.012

[11] Carroll EA, Schweppe M, Langfitt M, Miller AN, Halvorson JJ. Management of humeral shaft fractures. J Am Acad Orthop Surg. 2012;20(7):423–33. 22751161 10.5435/JAAOS-20-07-423

[12] Yang J, Yang Z, Liu D, Lu Z, Tao C, Liu T. Is an anteromedial minimally invasive approach for middle and distal third humeral fractures feasible? a cadaveric study and clinical case series. J Orthop Traumatol. 2023;24(1):7. 36764964 10.1186/s10195-023-00684-9 PMC9918646

[13] Liu D, Liang J, Yang H, Zhang Y, Lu Z. Medial minimally invasive percutaneous plate osteosynthesis for humeral shaft fractures: a case series and novel technique description. Arch Orthop Trauma Surg. 2023;143(11):6657–64. 37530845 10.1007/s00402-023-04992-x

[14] Huang Q, Lu Y, Wang ZM, Sun L, Ma T, Wang Q, et al. Anterolateral approach with two incisions versus posterior median approach in the treatment of middle- and distal-third humeral shaft fractures. J Orthop Surg Res. 2021;16(1):197. 33731159 10.1186/s13018-021-02355-z PMC7967943

[15] Oliver WM, Molyneux SG, White TO, Clement ND, Duckworth AD, Keating JF. Open reduction and internal fixation for humeral shaft nonunion: bone grafting is not routinely required and avoids donor site morbidity. J Orthop Trauma. 2021;35(8):414–23. 34267148 10.1097/BOT.0000000000002032

[16] Adams J. Fractures of the shaft of the humerus: an epidemiological study of 401 fractures. Yearbook of Hand and Upper Limb Surgery. 2008;2008:9–10. 10.1016/S1551-7977(08)79110-6

[17] Noble J, Munro CA, Prasad VS, Midha R. Analysis of upper and lower extremity peripheral nerve injuries in a population of patients with multiple injuries. J Trauma. 1998;45(1):116–22. 9680023 10.1097/00005373-199807000-00025

[18] Amillo S, Barrios RH, Martínez-Peric R, Losada JI. Surgical treatment of the radial nerve lesions associated with fractures of the humerus. J Orthop Trauma. 1993;7(3):211–5. 8326423 10.1097/00005131-199306000-00002

[19] Mast JW, Spiegel PG, Harvey JP, Harrison C. Fractures of the humeral shaft: a retrospective study of 240 adult fractures. Clin Orthop Relat Res. 1975;112:254–62. 10.1097/00003086-197510000-00033 1192642

[20] Holstein A, Lewis GM. Fractures of the humerus with radial-nerve paralysis. J Bone Joint Surg Am. 1963;45:1382–8. 14069777 10.2106/00004623-196345070-00004

[21] Shao YC, Harwood P, Grotz MRW, Limb D, Giannoudis PV. Radial nerve palsy associated with fractures of the shaft of the humerus: a systematic review. J Bone Joint Surg Br. 2005;87(12):1647–52. 16326879 10.1302/0301-620X.87B12.16132

[22] Ljungquist KL, Martineau P, Allan C. Radial nerve injuries. J Hand Surg Am. 2015;40(1):166–72. 25442768 10.1016/j.jhsa.2014.05.010

[23] Streufert BD, Eaford I, Sellers TR, Christensen JT, Maxson B, Infante A, et al. Iatrogenic nerve palsy occurs with anterior and posterior approaches for humeral shaft fixation. J Orthop Trauma. 2020;34(3):163–8. 31842186 10.1097/BOT.0000000000001658

[24] Laulan J. High radial nerve palsy. Hand Surg Rehabil. 2019;38(1):2–13. 30528552 10.1016/j.hansur.2018.10.243

[25] Bumbasirevic M, Palibrk T, Lesic A, Atkinson HD. Radial nerve palsy. EFORT Open Rev. 2016;1(8):286–94. 28461960 10.1302/2058-5241.1.000028 PMC5367587

[26] Ouyang H, Xiong J, Xiang P, Cui Z, Chen L, Yu B. Plate versus intramedullary nail fixation in the treatment of humeral shaft fractures: an updated meta-analysis. J Shoulder Elbow Surg. 2013;22(3):387–95. 22947239 10.1016/j.jse.2012.06.007

[27] Kurup H, Hossain M, Andrew JG. Dynamic compression plating versus locked intramedullary nailing for humeral shaft fractures in adults. Cochrane Database Syst Rev. 2011. 10.1002/14651858.CD005959.pub2. 21678350 10.1002/14651858.CD005959.pub2 PMC12597225

[28] Martínez AA, Cuenca J, Herrera A. Treatment of humeral shaft nonunions: nailing versus plating. Arch Orthop Trauma Surg. 2004;124(2):92–5. 14652778 10.1007/s00402-003-0608-7

[29] Singh AK, Arun GR, Narsaria N, Srivastava A. Treatment of non-union of humerus diaphyseal fractures: a prospective study comparing interlocking nail and locking compression plate. Arch Orthop Trauma Surg. 2014;134(7):947–53. 24853958 10.1007/s00402-014-1973-0

[30] Koh J, Tornetta P, Walker B, Jones C, Sharmaa T, Sems S, et al. What is the real rate of radial nerve injury after humeral nonunion surgery? J Orthop Trauma. 2020;34(8):441–6. 32569074 10.1097/BOT.0000000000001755

[31] Olarte CM, Darowish M, Ziran BH. Radial nerve transposition with humeral fracture fixation: preliminary results. Clin Orthop Relat Res. 2003;413:170–4. 10.1097/01.blo.0000072470.32680.60 12897607

[32] Chamseddine AH, Abdallah A, Zein H, Taha A. Transfracture medial transposition of the radial nerve associated with plate fixation of the humerus. Int Orthop. 2017;41(7):1463–70. 28105502 10.1007/s00264-016-3397-7

[33] Zalavras CG, Yasmeh S, Bougioukli S. Surgical management of humeral shaft nonunions. success of a consistent protocol over 17 years. Injury. 2021;52(12):3580–7. 33933273 10.1016/j.injury.2021.04.046

[34] Nicolaci G, Maes V, Lollino N, Putzeys G. How to treat proximal and middle one-third humeral shaft fractures: the role of helical plates. Musculoskelet Surg. 2023;107(2):231–8. 35579822 10.1007/s12306-022-00748-9

[35] Bryan RS, Morrey BF. Extensive posterior exposure of the elbow. a triceps-sparing approach. Clin Orthop Relat Res. 1982;166:188–92. 10.1097/00003086-198206000-00033 7083671

[36] Gerwin M, Hotchkiss RN, Weiland AJ. Alternative operative exposures of the posterior aspect of the humeral diaphysis with reference to the radial nerve. J Bone Joint Surg Am. 1996;78(11):1690–5. 8934483 10.2106/00004623-199611000-00008

[37] O’Driscoll SW. The triceps-reflecting anconeus pedicle (TRAP) approach for distal humeral fractures and nonunions. Orthop Clin North Am. 2000;31(1):91–101. 10629335 10.1016/S0030-5898(05)70130-9

[38] Shoji K, Heng M, Harris MB, Appleton PT, Vrahas MS, Weaver MJ. Time from injury to surgical fixation of diaphyseal humerus fractures is not associated with an increased risk of iatrogenic radial nerve palsy. J Orthop Trauma. 2017;31(9):491–6. 28459772 10.1097/BOT.0000000000000875

[39] Shon HC, Yang JY, Lee Y, Cho JW, Oh JK, Lim EJ. Iatrogenic radial nerve palsy in the surgical treatment of humerus shaft fracture -anterolateral versus posterior approach: a systematic review and meta-analysis. J Orthop Sci. 2023;28(1):244–50. 34716068 10.1016/j.jos.2021.09.015

[40] Tetsworth K, Hohmann E, Glatt V. Minimally invasive plate osteosynthesis of humeral shaft fractures: current state of the art. J Am Acad Orthop Surg. 2018;26(18):652–61. 30113346 10.5435/JAAOS-D-17-00238

[41] Feng YuB, le Liu L, Guo-jing Y, Zhang L, Xi-peng L. Comparison of minimally invasive plate osteosynthesis and conventional plate osteosynthesis for humeral shaft fracture: a meta-analysis. Medicine. 2016;95(39): e4955. 27684839 10.1097/MD.0000000000004955 PMC5265932

[42] Hu X, Xu S, Lu H, Chen B, Zhou X, He X, et al. Minimally invasive plate osteosynthesis vs conventional fixation techniques for surgically treated humeral shaft fractures: a meta-analysis. J Orthop Surg Res. 2016;11(1):59. 27169580 10.1186/s13018-016-0394-x PMC4864922

[43] Zhao JG, Wang J, Meng XH, Zeng XT, Kan SL. Surgical interventions to treat humerus shaft fractures: a network meta-analysis of randomized controlled trials. PLoS ONE. 2017;12(3): e0173634. 28333947 10.1371/journal.pone.0173634 PMC5363833

[44] Li WY, Zhang BS, Zhang L, Zheng SH, Wang SM. Comparative study of antegrade and retrograde intramedullary nailing for the treatment of humeral shaft fractures. Zhongguo Gu Shang. 2009;22(3):199–201. 19366103

[45] Wang Q, Hu J, Guan J, Chen Y, Wang L. Proximal third humeral shaft fractures fixed with long helical PHILOS plates in elderly patients: benefit of pre-contouring plates on a 3D-printed model-a retrospective study. J Orthop Surg Res. 2018;13(1):203. 30119637 10.1186/s13018-018-0908-9 PMC6098615

[46] Chirattikalwong S, Suwannaphisit S, Wuttimanop W, Chuaychoosakoon C. Risk of radial nerve injury in anterolateral humeral shaft plating. J Am Acad Orthop Surg. 2022;30(18):903–9. 36166385 10.5435/JAAOS-D-21-00970

[47] Chuaychoosakoon C, Chirattikalwong S, Wuttimanop W, Boonriong T, Parinyakhup W, Suwannaphisit S. The risk of iatrogenic radial nerve and/or profunda brachii artery injury in anterolateral humeral plating using a 45 mm narrow DCP: a cadaveric study. PLoS ONE. 2021;16(11): e0260448. 34847166 10.1371/journal.pone.0260448 PMC8631653

[48] Noger M, Berli MC, Fasel JHD, Hoffmeyer PJ. The risk of injury to neurovascular structures from distal locking screws of the Unreamed Humeral Nail (UHN): a cadaveric study. Injury. 2007;38(8):954–7. 17631884 10.1016/j.injury.2007.04.014

[49] von der Helm F, Fenwick A, Reuter J, Adolf-Lisitano L, Mayr E, Förch S. New ways of treatment of fractures of the humeral shaft: does the combination of intramedullary nail osteosynthesis and cerclage improve the healing process? Eur J Trauma Emerg Surg. 2022;48(4):3081–7. 34971422 10.1007/s00068-021-01847-1 PMC9360159

